# Applications and Major Achievements of Genome Editing in Vegetable Crops: A Review

**DOI:** 10.3389/fpls.2021.688980

**Published:** 2021-06-11

**Authors:** Young-Cheon Kim, Yeeun Kang, Eun-Young Yang, Myeong-Cheoul Cho, Roland Schafleitner, Jeong Hwan Lee, Seonghoe Jang

**Affiliations:** ^1^Division of Life Sciences, Jeonbuk National University, Jeonju, South Korea; ^2^World Vegetable Center Korea Office, Wanju-gun, South Korea; ^3^National Institute of Horticultural and Herbal Science (NIHHS), Rural Development Administration (RDA), Wanju-gun, South Korea; ^4^World Vegetable Center, Tainan, Taiwan

**Keywords:** CRISPR-Cas application, genome-editing technology, precision breeding, transformation, vegetables

## Abstract

The emergence of genome-editing technology has allowed manipulation of DNA sequences in genomes to precisely remove or replace specific sequences in organisms resulting in targeted mutations. In plants, genome editing is an attractive method to alter gene functions to generate improved crop varieties. Genome editing is thought to be simple to use and has a lower risk of off-target effects compared to classical mutation breeding. Furthermore, genome-editing technology tools can also be applied directly to crops that contain complex genomes and/or are not easily bred using traditional methods. Currently, highly versatile genome-editing tools for precise and predictable editing of almost any locus in the plant genome make it possible to extend the range of application, including functional genomics research and molecular crop breeding. Vegetables are essential nutrient sources for humans and provide vitamins, minerals, and fiber to diets, thereby contributing to human health. In this review, we provide an overview of the brief history of genome-editing technologies and the components of genome-editing tool boxes, and illustrate basic modes of operation in representative systems. We describe the current and potential practical application of genome editing for the development of improved nutritious vegetables and present several case studies demonstrating the potential of the technology. Finally, we highlight future directions and challenges in applying genome-editing systems to vegetable crops for research and product development.

## Introduction

There has been growing interest in the beneficial effect of consuming vegetables because of the broad range of nutritional compounds, such as vitamins, minerals, antioxidants, dietary fiber, and a plethora of phytochemical compounds present in this crop group (Septembre-Malaterre et al., 2018). While vitamins and minerals are essential nutrients for humans, antioxidant compounds from fruits and vegetables are known to reduce cellular oxidative stress and the risk of chronic disease, like diabetes, cancer, and cardiovascular disease (Serafini et al., 2002; Ninfali et al., 2005; Aune et al., 2017; Miller et al., 2017). However, vegetables, like all other crops, are generally sensitive to biotic and abiotic stresses, and thus, disease, high temperature, and limited water supply are major limiting factors in vegetable productivity. These factors will be further magnified by climate change (Bisbis et al., 2018). Therefore, researchers are striving to improve vegetable varieties in terms of yield and yield stability, nutritional value, and biotic and abiotic stress tolerance by classical breeding and by using plant molecular breeding technologies (Abdallah et al., 2015).

Plant breeding is a complex process through which new crop varieties with desirable characteristics are developed and strategies are devised to combine these characteristics to obtain superior varieties (Glenn et al., 2017). The first step in breeding is to make use of genetic variation between individuals within a plant species. For decades, this has been achieved by crossing parental material or by applying physical or chemical mutagenesis to crop plants (Holme et al., 2019; Kleter et al., 2019). Since pioneering research at the beginning of the twentieth century detected the capacity of X-rays to change the plant phenotype in crops, such as barley and maize, a series of novel mutagenesis breeding tools have been developed (Muller, 1927; Stadler, 1928a,b). More sophisticated radiation techniques, such as gamma radiation, UV light, and particle radiation, were then developed to generate novel agronomic traits in crops and to study gene functions (Shu et al., 2012). Later, mutagenic chemicals were preferred for mutagenesis because of easier handling and higher mutation frequency compared to radiological methods. Today, the most widely used chemical mutagen is ethyl methanesulfonate, with other chemical mutagens, such as sodium azide and methyl nitrosourea (Az-MNU), also in frequent use for mutagenesis in plants (Till et al., 2004, 2007).

Physical and chemical mutagenesis has been successfully employed to induce point mutations and deletions in the plant genome (Jankowicz-Cieslak et al., 2017), and thousands of varieties, derived from mutation breeding, have been released (Ahloowalia et al., 2004). The promise of mutagenesis in vegetable breeding has remained largely unfulfilled, but it is widely used to generate variation in ornamentals (Bernardo, 2016). As molecular technologies and DNA sequencing technologies advanced, researchers began to study the functional characteristics of genes by using T-DNA-tagged mutant pools generated by random T-DNA insertional mutagenesis, enabling identification of DNA sequences flanking T-DNA to explore potential genetic elements responsible for phenotypic alterations in mutants. The information accumulated from the analyses of T-DNA flanking sequences is one of the stepping stones that provided new pathways for producing improved crop varieties harboring desired traits with the aid of molecular breeding tools (Radhamony et al., 2005; Chaudhary et al., 2019; Kleter et al., 2019).

There have been a number of major advances in molecular biological methods over the last few decades. The discovery of sequence-specific nucleases (SSNs) and the development of the clustered regulatory interspaced short palindromic repeats (CRISPR)/CRISPR-associated protein (Cas) system have enabled programmable gene editing at the DNA level to generate vegetables with altered functions and desired traits (Abdallah et al., 2015; Nunez de Caceres Gonzalez and De la Mora Franco, 2020).

In this review, we describe the brief history of genome editing, the components of the genome-editing tool boxes, and basic modes of gene-editing systems. In addition, we present examples of practical application of those tools in editing vegetable genomes. Finally, future directions and challenges associated with genome-editing systems for the production of vegetables with desired traits are discussed.

## History of Genome-/Gene-Editing Technology

**Figure 1:**
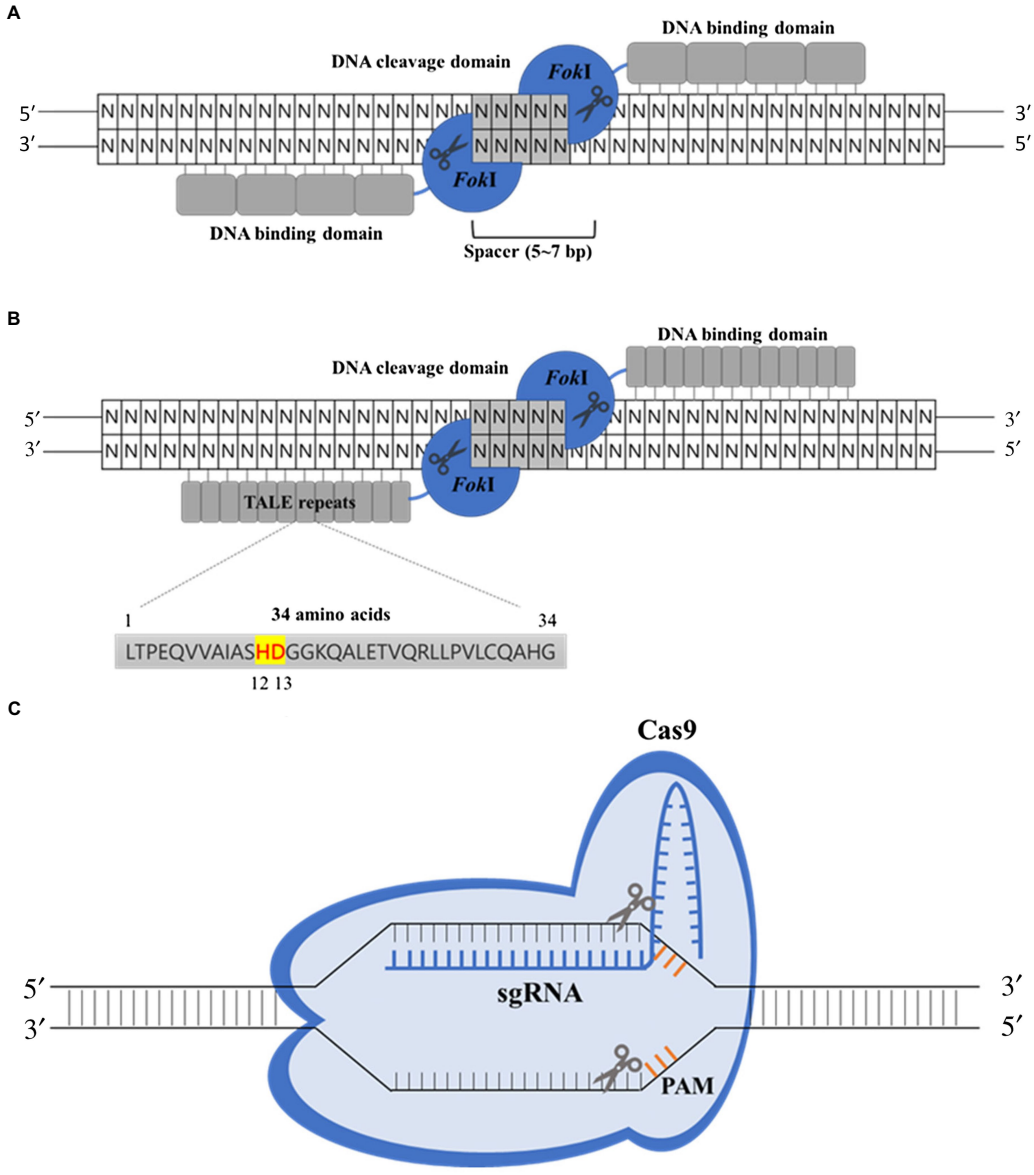
Some of the major genome-editing technologies using site-specific nucleases include zinc-finger nucleases (ZFNs), transcription activator-like effector nucleases (TALENs), and the clustered regularly interspaced short palindromic repeats/CRISPR-associated 9 (CRISPR-Cas9) systems. **(A)** The ZFN-binding domain is comprised of multimerized zinc-finger proteins (ZFPs). Each ZFP recognizes approximately 3 bp of DNA sequence, and the fused *Fok*I nuclease domains dimerize and generate double-strand breaks. **(B)** Like ZFNs, TALENs consist of a DNA-binding domain, termed transcription activator-like effector (TALE) repeats, and nuclease domain of *Fok*I enzyme. Each TALE repeat consists of a highly conserved 34-amino acid sequence with hypervariable twelfth and thirteenth amino acids, which allow the recognition of the single nucleotide. **(C)** In the CRISPR-Cas9 system, a single-guide RNA (sgRNA) guides the Cas9 nuclease to direct the cleavage of cognate DNA sequences adjacent to 5'-NGG-3' protospacer-adjacent motifs (PAMs).

Over the past few years, the development of gene-/genome-editing technologies has facilitated the precise and efficient targeted modification of genomes in various organisms, including vegetable crops to increase yield and quality (Chen and Gao, 2014). Advanced molecular biological methods using SSNs, such as zinc-finger nucleases (ZFNs) and transcription activator-like effector nucleases (TALENs), and CRISPR-Cas system (Kim et al., 1996; Christian et al., 2010; Jinek et al., 2012; Chen and Gao, 2014; Gao, 2014), have made it possible for plant researchers to conduct targeted gene/genome engineering precisely and efficiently. These techniques generate double-strand breaks (DSBs) at specific DNA sites and, via the endogenous DNA repair system, induce insertions or deletions of nucleotides by non-homologous end joining (NHEJ), or cause gene replacements by homologous recombination (HR) thereby resulting in loss-of-function or gain-of-function of target genes (Symington and Gautier, 2011; Figure 1). The various CRISPR-Cas systems have been revealed to be powerful tools for gene/genome editing, and numerous genome-edited plants have been created by these gene-/genome-editing technologies (Gao, 2014).

### Zinc-Finger Nucleases

Zinc-finger nucleases, the first generation of site-specific nucleases, allowed the rapid and targeted modification of the genome (Kim et al., 1996). ZFNs are typically generated by fusing zinc-finger protein (ZFP) domains and are capable of sequence-specific DNA binding, with a nonspecific DNA cleavage domain from bacterial *Fok*I endonuclease (Figure 1A; Petolino, 2015). Each ZFP with a tandem array of cysteine2 and histidine2 (Cys_2_-His_2_) domains (Miller et al., 1985) recognizes approximately 3 bp of DNA sequence. Generally, four to six ZFPs are linked together to recognize a specific DNA sequence (12–18 bp; Urnov et al., 2010). The *Fok*I catalytic domain must dimerize for DNA cleavage (Bitinaite et al., 1998), so ZFNs are used as heterodimers to target and cut the DNA. Once the zinc-finger domain recognizes and binds the target DNA, the fused *Fok*I nuclease domain cleaves the DNA sequence, inducing DSBs at their target locus (Weinthal et al., 2010; Lee et al., 2016).

Zinc-finger nucleases have been employed to modify the targeted gene sequence in several crops, such as maize (Shukla et al., 2009; Ainley et al., 2013), soybean (Curtin et al., 2011), rice (Cantos et al., 2014; Jung et al., 2018), and apple (Peer et al., 2015). As a representative example of the genome editing in crops by ZFNs, Shukla et al. (2009) reported the precise targeted insertion of the *PAT* herbicide-tolerance gene at the *IPK1* locus in maize (Shukla et al., 2009). These mutated maize plants exhibited herbicide tolerance as well as altered inositol phosphate profiles in the developing seed. ZFNs were also used for the elucidation of functional roles of the *SSIVa* encoding an isoform of soluble starch synthase in rice starch biosynthesis (Jung et al., 2018). Transgenic plants containing ZFN-induced mutations at the *SSIVa* locus showed dwarfism with reduced starch content. The modularity of ZFNs is a major advantage for the design of DNA-binding proteins that can recognize a broad spectrum of DNA sequences for genome editing (Bhakta et al., 2013). Despite the advantage of ZFNs in genome editing, several inherent disadvantages have restricted their wide range of application, such as expensive and time-consuming processes for optimized assembly of the ZF domain to bind DNA with high affinity and low frequency in selection of target sites, which can only be used every 200 bps in DNA sequences and occurrence of imperfect modular structure in zinc-finger assembly and/or nonspecific binding of the *Fok*I cleavage domain, which can raise the risk of off-target effects and/or cellular toxicity (Eid and Mahfouz, 2016; Mushtaq et al., 2018).

### Transcription Activator-Like Effector Nucleases

Transcription activator-like effector nucleases are the second generation of site-specific nucleases, which have emerged as an alternative to ZFNs for genome editing (Sakuma and Yamamoto, 2017). TALENs consist of DNA-binding and nuclease domains of *Fok*I enzyme like ZFNs. The DNA-binding domain, termed transcription activator-like effector (TALE) repeats, is typically composed of repetitive sequences of residues derived from TALEs secreted by *Xanthomonas* bacteria to aid the infection of plant species (Figure 1B). Each DNA-binding repeat recognizes a single nucleotide of the genomic sequence (Boch and Bonas, 2010; Joung and Sander, 2013). An individual DNA-binding repeat consists of a highly conserved 34-amino acid sequence with a highly polymorphic region at positions 12 and 13, which are referred to as the repeat variable di-residue (RVD) determining the nucleotide-binding specificity (Richter et al., 2020). Therefore, a TALE DNA-binding domain with the nuclease domain of *Fok*I can generate DSBs at a desired target site in the genome by selecting a combination of RVDs. Like ZFNs, TALEN-mediated genome editing has achieved targeted mutagenesis in a variety of crop species, such as rice (Li et al., 2012; Shan et al., 2013), soybean (Haun et al., 2014), wheat (Wang et al., 2014), and potato (Sawai et al., 2014). Two seed-specific genes, *FAD2-1A* and *FAD2-1B* encoding a fatty acid desaturase-2 enzyme (FAD2-1) that converts oleic acid to linoleic acid in soybean, were deleted to improve the quality of soybean seed oil by TALENs system (Haun et al., 2014). The genetically engineered soybean plants produced nearly four times more oleic acid than the wild-type (WT) parents. In addition, Wang et al. (2014) successfully utilized the TALENs genome-editing system in wheat to conduct the targeted manipulation of three *MLO* loci, which encode proteins that repress defenses against powdery mildew diseases in other plants: TALEN-induced *tamlo-aabbdd* wheat plants showed strong resistance to powdery mildew (Wang et al., 2014).

A TALE repeat module recognizes a single nucleotide, while a single zinc-finger module recognizes three nucleotides of DNA (Jankele and Svoboda, 2014), so the design of the TALEN genome-editing machinery is more facile and adaptable compared to ZFNs. In addition, in contrast to ZFNs, the TALE repeat array can be extended to any desired length, though the use of larger TALENs may result in less specificity (Gupta and Musunuru, 2014). Although TALEN has the advantage of greater flexibility, it is sensitive to cytosine methylation especially at CpG dinucleotides (Valton et al., 2012), and to be active, it requires thymine before the 5' end of the target sequence, which is recognized by two amino-terminal cryptic repeat folds (Kim and Kim, 2014).

### CRISPR-Cas9

Even though both ZFN and TALEN have been proven to be quite effective for gene editing, these tools are relatively expensive and laborious to engineer, which has restricted their application (Gaj et al., 2013; Erpen-Dalla Corte et al., 2019). DNA/RNA-mediated adaptive immune systems involving type II CRISPR and Cas immunity in bacteria and archaea that protect against invading plasmids and viruses provide an alternative genome-editing strategy (Barrangou, 2013). In this system, when foreign DNA from a virus or plasmid is integrated into a CRISPR locus, the CRISPR repeat-spacer array is converted into the mature CRISPR RNA (crRNA). The mature crRNA that is base-paired to trans-activating crRNA (tracrRNA) guides the CRISPR-associated endonuclease, Cas9 to cleave the protospacer DNA on both strands of the invader (Horvath and Barrangou, 2010; Jinek et al., 2012). The CRISPR-Cas9 mechanism has emerged as a powerful universal genome-editing tool, also for targeted trait improvement in crops (Arora and Narula, 2017).

The CRISPR-Cas9 system for genome editing has two components: the Cas9 nuclease and a single-guide RNA (sgRNA) consisting of the artificial fusion of a crRNA and the scaffold tracrRNA (Figure 1C; Jinek et al., 2012). The sgRNA guides Cas9 to direct the cleavage of cognate DNA sequences predominantly 3 bp away from the 5'-NGG-3' protospacer-adjacent motif (PAM; Fischer et al., 2012). Therefore, a variety of DNA sites can be targeted by Cas9 through exchange of the 20-bp spacer sequence in the guide RNA (gRNA) with a sequence that is complementary to the target site (Cong et al., 2013). Major advantages of the CRISPR-Cas9 system over ZFNs and TALENs are simplicity and the low cost of engineering of the system, its adaptability to virtually to any target region with a PAM sequence nearby, and its capacity for multiplexing, meaning that multiple sites can be targeted for mutagenesis simultaneously by using multiple sgRNAs while expressing a single Cas9 nuclease gene (Pennisi, 2013; Minkenberg et al., 2017). However, there are still some challenges to be tackled to apply the CRISPR-Cas9 technology to the gene/genome editing of crops. Although the CRISPR-Cas9 system is more precise and efficient in gene/genome editing than other previously developed genetic engineering methods, there is a risk of off-target effects, which are defined as unintended cleavages and/or mutations at untargeted genomic sites (Zhao and Wolt, 2017). Off-target mutations may cause unintended effects, especially in species with large and complex genomes (Alkan et al., 2018). Methods for reducing the risk of off-target mutations have been developed, as discussed below. Furthermore, off-target effects in plants can be mitigated by selecting the desired phenotypes in the pool of mutated individuals or by breeding. CRISPR-Cas9 is thought to be particularly advantageous in plant breeding to change a single or a few alleles in a line, especially when the alternative would consist of introgressing the desired allele from non-adapted accessions or wild species. In these cases, CRISPR-Cas9-mediated gene editing avoids linkage drag and makes backcrossing to achieve the recurrent phenotype unnecessary. It allows producing specific mutations effectively in a selected genetic background.

## Precise Genome Editing in Plants

Targeted mutagenesis in plants relies on NHEJ to repair DSBs, which is error-prone and, at a certain frequency, results in small deletions, insertions, or nucleotide substitutions (Atkins and Voytas, 2020). Homology-directed repair (HDR)-mediated repair is less common than the NHEJ repair pathway due to very low editing efficiency achieved with HDR, but allows targeted gene insertions or replacement, if a repair template is provided. The modern mutation tools, including ZNFs, TALENs, and CRISPR-Cas systems, have revolutionized the field of plant molecular biology and functional genomics research and have the potential to facilitate crop development by altering agronomic traits (Chen et al., 2019). Although the ZNF and TALEN systems have contributed to the targeted mutagenesis studies in a variety of plant species, such as *Arabidopsis*, maize, *Brassica*, rice, barley, soybean, tobacco, tomato, wheat, potato, and sugarcane (Shelake et al., 2019), the CRISPR-Cas system is now the most widely adopted tool for genome editing due to its simple engineering process, versatility, low cost, high efficiency, and high specificity (Brandt and Barrangou, 2019) and has great potential for genome editing to develop value-added improved cultivars (Zhang et al., 2020c). In the following sections, we provide an overview of the most commonly used and the newly developed CRISPR-Cas9 technologies for precise genome editing in plants.

### Basic CRISPR-Cas System

**Figure 2:**
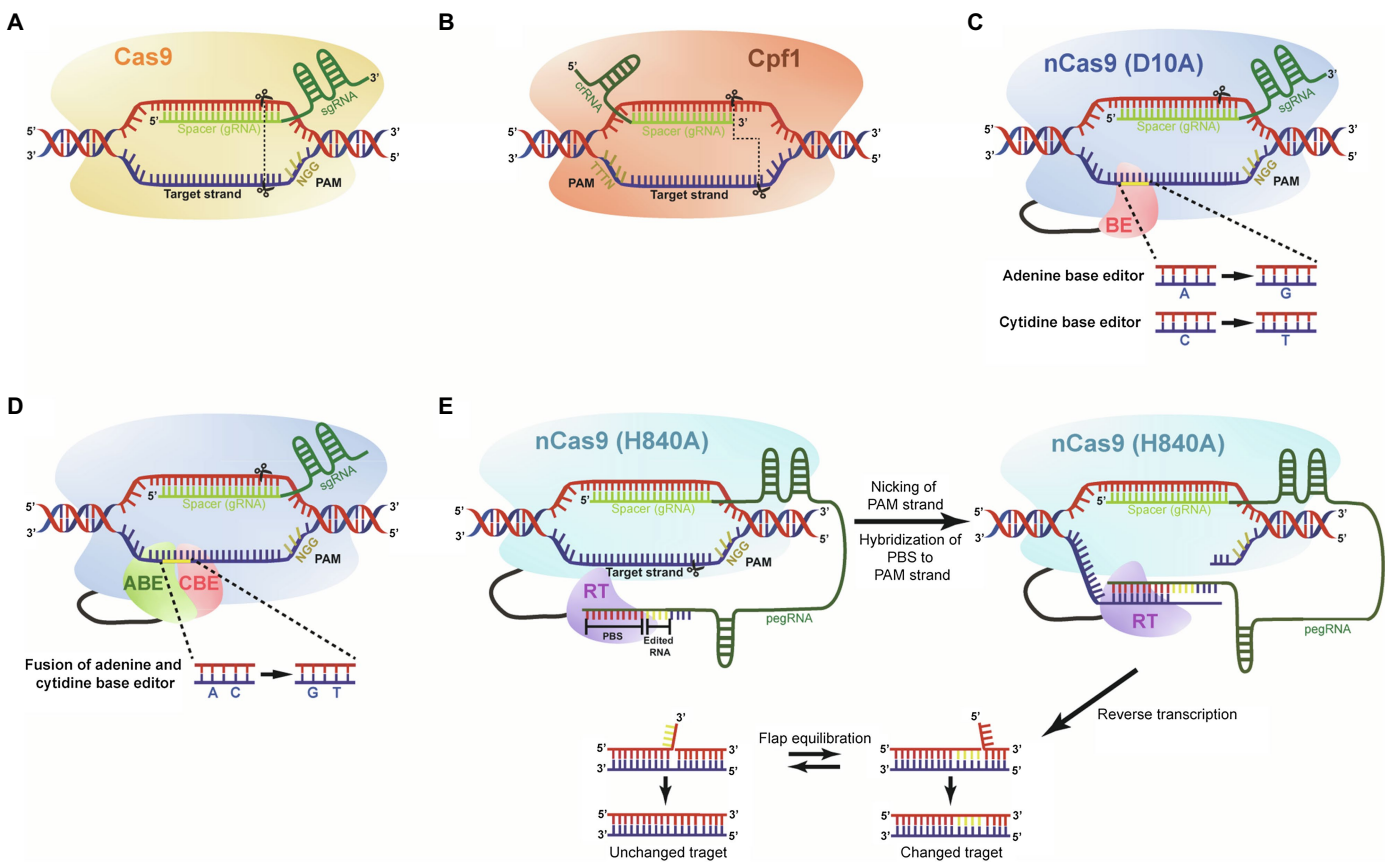
The main CRISPR-Cas-mediated genome-editing systems. **(A)** Diagram of the CRISPR-Cas9 is shown with a sgRNA encoding a spacer guide RNA (gRNA) positioned next to PAM (5'-NGG) site. Cleavage sites by Cas9 protein are shown with scissors, and blunt ends are presented with dotted line. **(B)** A diagram of the CRISPR-Cpf1 is shown with crRNA encoding a spacer gRNA positioned next to PAM (5'-TTTTN) site. Cleavage sites by Cpf1 protein are shown with scissors, and staggered ends with 5' overhang are presented with dotted line. **(C)** Base editor (BE) composed of nCas9 nickase (D10A). The base editing system has two versions: adenine and cytidine BEs converting A to G and C to T, respectively. **(D)** nCas9 nickase (D10A) fused with adenine base editor and cytidine base editor generates A to G and C to T substitutions, simultaneously. **(E)** Prime editor composed of nCa9 nickase (H840A), reverse transcriptase (RT), and prime editing guide RNA (pegRNA). The pegRNA carries spacer, desired editing sequence, and primer binding site (PBS). The PBS binds to the nicked strand, and then, RT copies sequences from the template. Flap equilibration results in unchanged and mutated DNA strands.

The CRISPR-Cas9 system has been widely adopted as a genome engineering platform (Figure 2A). Cas9 (SpCas9), the central player in the type II CRISPR-Cas Class2 system of *Streptococcus pyogenes*, was the first nuclease harnessed for genome editing and is still the most commonly used enzyme for this purpose (Cong et al., 2013; Mali et al., 2013). A sgRNA is designed to function in the crRNA-tracrRNA complex. Cas9 protein can be targeted to specific genomic loci by sgRNAs with various sequences: Cas9 recognizes sgRNAs and is then guided to bind a complementary sequence at the target sites, which is positioned next to PAM by hybridization of the spacer part of the sgRNAs. Subsequently, the CRISPR-Cas9 system breaks both strands of the DNA, resulting in blunt end cuts generally three base pairs upstream (−3 bp) of the PAM site (5'-NGG; Gasiunas et al., 2012; Jinek et al., 2012; Makarova et al., 2015).

To expand the limited availability of SpCas9 target sites by recognizing different PAM sequences, additional SpCas9 variants have been developed, such as xCas9 recognizing 5'-NG, 5'-GAA, and 5'-GAT (Hu et al., 2018), *Staphylococcus aureus* Cas9 (SaCas9) recognizing 5'-NNGRRT (Ran et al., 2015), *S. thermophilus* Cas9 (StCas9) recognizing 5'-NNAGAAW (Cong et al., 2013), *Neisseria meningitidis* Cas9 (NmCas9) recognizing 5'-NNNNGATT (Esvelt et al., 2013), *Campylobacter jejuni* Cas9 (CjCas9) recognizing 5'-NNNVRYM (Yamada et al., 2017), and CasX known as Cas12e recognizing 5'-TTCN (Burstein et al., 2017). Cas13 variants mediating high interference activities against target RNA viruses have been also applied to plants for studying virus biology (Mahas et al., 2019).

After a DSB is inserted at the target site by CRISPR-Cas9, DNA repair occurs via NHEJ, which often causes mutations, such as an insertion or more often a deletion at the repair site. If this mutation is in a genic region, such indels very likely generate a premature stop codon downstream of the target sites (Cong et al., 2013; Mali et al., 2013). Cpf1 known as Cas12a, another Class 2 endonuclease, is similar to Cas9 in size and shape, and recognizes 5'-TTTTN or TTTN as the PAM sequence (Zetsche et al., 2015). However, functional Cpf1 does not require tracrRNA – but still needs gRNA providing specificity. In addition, Cpf1 generates a staggered cut with a 5' overhang at the target sites, which may be beneficial for the correct orientation of integrating DNA (Figure 2B). Furthermore, AsCpf1, a Cpf1 orthologue from *Acidaminococcus* spp., recognizes the 5'TTTV as a PAM sequence and their variants were recently engineered to increase genome-editing activity and expand PAM recognition sites by recognizing 5'-TYCV and 5'-TATV or 5'-VTTV, 5'-TTTT, 5'-TTCN, and TATV (Gao et al., 2017; Kleinstiver et al., 2019).

### Base Editing

Deaminase-mediated base editing technology is an alternative genome-editing tool, which can generate precise point mutations in the target region of genomes without making DSBs. Instead, a base editor is fused to a Cas9 nickase and is targeted to a specific DNA sequence with a gRNA. After the advent of two primary base editing tools, cytosine base editors (CBEs) and adenine base editors (ABEs; Shimatani et al., 2017; Li et al., 2020b), dual base editor-mediated precise genome-editing technology was developed in plants (Li et al., 2020b). CBE consists of a Cas9 nickase (nCas9) harboring the D10A mutation that inactivates the RuvC, one of the two nucleolytic domains (RuvC and HNH) of Cas9, fused with a cytidine deaminase and uracil glycosylase inhibitor (UGI; Figure 2C). Deamination on cytidine catalyzed by cytidine deaminase in the targeting strand DNA region converts the cytidine into uridine. When nCas9 (D10A) protein nicks the nontargeting DNA strand, the induced U-G mismatch generates T-A in the resulting DNA strands through the DNA repair and replication processes (C-to-T). CBE-mediated base editing technology has been used in various crops, such as rice, maize, wheat, and potato with relatively high efficiency (Shimatani et al., 2017; Zong et al., 2017; Ren et al., 2018; Zong et al., 2018; Jin et al., 2020).

Adenine base editor, another base editing technology, expands base editing by converting A-T to G-C. The deamination of adenosine by adenosine deaminase fused with nCas9 (D10A) yields inosine, which can be paired with cytidine and is also recognized as guanine by DNA polymerase during DNA repair and replication (Gaudelli et al., 2017). Although ABE variants were developed in *Arabidopsis* (Kang et al., 2018), wheat, rice, and rapeseed (Li et al., 2018a), the system showed lower efficiency than the original SpCas9 or SaCas9 (Hua et al., 2019; Li et al., 2020b). Several ABE variants recently developed for adenine base editing in mammalian cells may be useful for efficient editing in plants (Gaudelli et al., 2020; Richter et al., 2020).

Dual base editing technology converting C-G to T-A and A-T to G-C simultaneously is based on the fusion of nCas9 with the two base editors mentioned above, which is referred to as saturated targeted endogenous mutagenesis editor (STEME). It generates simultaneous dual base substitutions (C-G to T-A and A-T to G-C) with a sgRNA, which was recently used for genome editing in rice (Li et al., 2020b). The STEME system composed of an ABE and a CBE, nCas9 (D10A), sgRNA, and a UGI facilitates directed evolution of endogenous genes (Figure 2D; Li et al., 2020b). Given that STEMEs can generate diverse mutations, including base substitutions and in-frame indels with high efficiency, dual base editing might be applicable for a study on *cis*-elements of noncoding regions and even genome-wide screening of *cis*-regulatory regions.

### Prime Editing

One of the major limitations of current genome-editing tools is in the technical difficulty with extremely low efficiency for introduction of customized sequences at target sites. Anzalone et al. (2019) developed the revolutionary genome-editing technology known as prime editing to solve this challenge (Anzalone et al., 2019). Prime editing enables 12 kinds of base conversions to target genes at locations ranging from 3 bp upstream to 29 bp downstream of PAM, consisting of precise insertions of up to 44 bp, and deletions of up to 80 bp without inducing DSBs (Anzalone et al., 2019). The prime editing system is composed of a nCas9 (H840A) fused to a reverse transcriptase (RTase) and a prime editing guide RNA (pegRNA). The pegRNA driving nCas9 (H840A) to bind the target sequence is comprised of two regions: One is the spacer complementary to the sequences of the nonedited DNA strand at 5' of the pegRNA and the other region is located at the 3' of the pegRNA containing prime binding site (PBS) required for the recognition of the sequences of DNA strand to be edited and the desired sequences, which will be introduced into the target site (Figure 2E). The PBS region plays a role as a primer for the RTase linked to the nCas9 (H840A). The RTase uses the pegRNA as the template, which pairs with the nCas9 (H840A)-nicked ssDNA strand, thereby resulting in a direct copy of the genetic information from the pegRNA into the target genome site (Anzalone et al., 2019). After the reverse transcription, the equilibration between 3' edited DNA flap and 5' unedited DNA flap occurs followed by integration of edited DNA into the target site of the genome via ligation and DNA repair system (Anzalone et al., 2019). Despite the prime editing system being a powerful genome-editing tool enabling the generation of 12 base substitutions and indels in rice and wheat, their practical efficiency in plants is limited, and also its capability for precise gene editing, up to now, was verified only in the two food crops: rice and wheat (Lin et al., 2020). Thus, more case studies demonstrating the applicability of the prime editing technology in various organisms are needed to accelerate the use of prime editing tools, and make steady progress toward technology improvement.

### Chromosome Engineering

The aim of breeding is to combine as many favorable traits as possible in a breeding line. Traits are conditioned by genes, and the genes are located on chromosomes. *Genes located* close to each other are almost always *inherited together*, a relationship referred to as complete linkage. Sometimes, target genes can be tightly linked with unfavorable traits and this linkage may be difficult to break through classical breeding. It was suggested that induction of cross overs (COs) to separate unfavorable linkages can be achieved by CRISPR-Cas9 and subsequent HR as a consequence of induced DSBs (Filler Hayut et al., 2017) although the frequency of these COs is low, and it requires a selection system that detects these HR events efficiently. Early studies showed that CRISPR-Cas9-mediated DSB induction enabled DNA fragment inversions in plants (Gao et al., 2015; Zhang et al., 2017) and CO suppression as large inversions could be eliminated successfully by reverting the inversion (Giraut et al., 2011), making the region again accessible to meiotic COs (Huang and Puchta, 2021). Introduction of multiple DSBs, with low efficiency, is also able to cause inversions (Schmidt et al., 2019). Schmidt et al. (2020) showed that a 1.1 Mb inversion on chromosome 4 of *Arabidopsis* could be reversed using SaCas9 for DSB induction (Schmidt et al., 2020). What is more, heritable CRISPR-Cas9-mediated reciprocal translocations in the Mbp range were obtained between different nonallelic chromosomes (Beying et al., 2020). This approach provides a new method for plant breeding: Introgression of genes from a wild relative in a cultivar may be strongly enhanced by reducing linkage drag and making lengthy backcrossing unnecessary.

## Delivery of CRISPR-Cas Agents in Plants

Through the rapid evolution of CRISPR-Cas tools with various functionalities, capabilities, and specialized application, CRISPR-Cas-mediated plant genome editing has become a very efficient and effective application in the area of crop improvement and translational research (Vats et al., 2019). However, there are bottlenecks in application of the editing technology to crop functional genomics and crop improvement. Researchers have focused on the delivery of gene-/genome-editing reagents to plant cells, which is one of the critical bottlenecks for successful plant gene/genome editing to produce the intended effects. The most commonly used delivery methods for DNA constructs carrying the CRISPR-cas9 components are biolistic bombardment, *Agrobacterium*-mediated delivery to explants or plants, and direct transfer of the constructs to protoplasts with polyethylene glycol (PEG), which have been successful for generating CRISPR-Cas-mediated genome editing in various plant species with varying efficiencies of transformation (Borrelli et al., 2018). However, these conventional delivery methods (plasmid-based transfer) have limitations, such as low efficiency and/or difficulty in transformation and regeneration depending on the plant species or genotype. Together with these limitations, conventional delivery methods also have controversy about safety concerns and legal restrictions on the use of recombinant DNA, warranting the development of DNA-free gene-editing methods.

**Figure 3:**
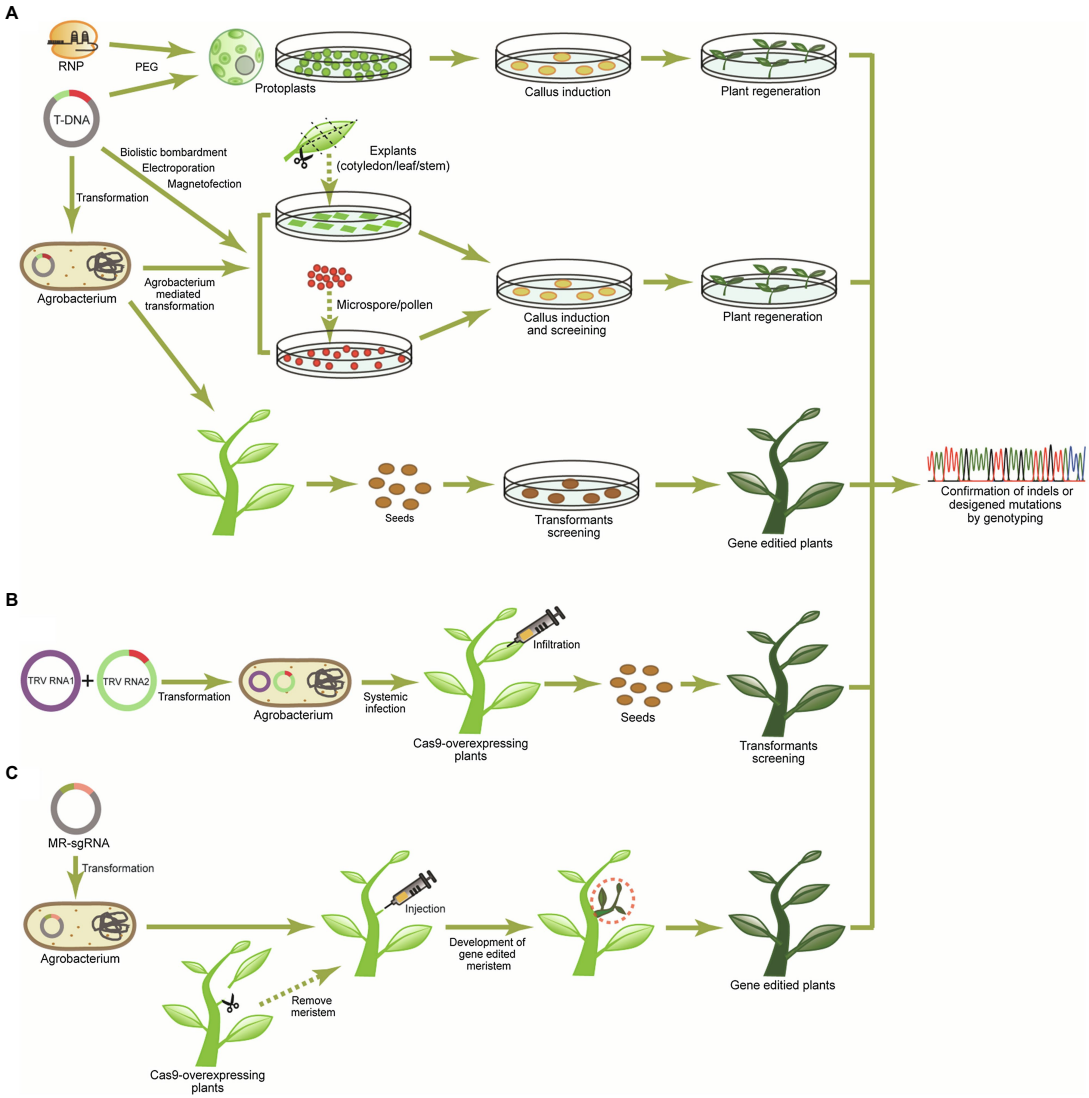
Strategies for delivery of the CRISPR-Cas system into plants. **(A)** The preassembled CRISPR-Cas9 ribonucleoproteins (RNPs) can be delivered into protoplasts through the polyethylene glycol (PEG)-mediation, and T-DNA encoding CRISPR-Cas reagents [Cas protein and sgRNA(s)] can be delivered into the rigid plan cells (explants, microspores/pollens, and intact plants) using *Agrobacterium*-mediated transformation, biolistic bombardment, and magnetofection. Subsequently, regeneration procedures of protoplasts and tissues carrying CRISPR-Cas reagents are needed to produce genome edited lines. **(B)** In the virus-induced gene-editing system, sgRNA fused with RNA mobile element is integrated into tobacco rattle virus (TRV) RNA2. After transformation of TRV RNA1 and TRV RNA2 to *Agrobacterium*, infiltration is conducted to Cas9-overexpressing plants resulting in systemic spreading of the sgRNA by the mobile elements and induction of mutagenesis. **(C)** For *de novo* meristem induction system, the meristems of Cas9-overexpressing plants are removed for infiltration, and then, *Agrobacterium*carrying morphogenic regulators (MRs) and sgRNA are injected into pruning sites. MRs induce the *de novo* gene-edited meristem, and the gene-edited plants can finally be obtained from newly developed shoots.

A delivery method of the preassembled CRISPR-Cas9 ribonucleoproteins (RNPs) into protoplasts has been considered as an approach for DNA-free genome editing (Woo et al., 2015; Lee et al., 2016). CRISPR-Cas9 RNP-mediated genome editing has been reported in *Arabidopsis*, tobacco, lettuce, rice, maize, and bread wheat (Woo et al., 2015; Svitashev et al., 2016; Liang et al., 2017). Nevertheless, after direct CRISPR-Cas9 RNP delivery to protoplasts by PEG-mediated transfection, the process of protoplast regeneration is laborious, sophisticated, and time consuming and it is highly challenging to obtain whole regenerated plants (Figure 3A).

Due to the limitations of delivering CRISPR-Cas9 riboprotein complexes to plant cells, *Agrobacterium*-mediated T-DNA transformation is one of the most widely used delivery methods, allowing T-DNA cassettes including CRISPR-Cas machinery to be transferred into intact plant cells or explants (Figure 3A). Although biolistic bombardment also can deliver T-DNA to plant cells, it is limited by low transformation efficiency and can cause chromosome damage and trigger a range of DNA rearrangement processes (Liu et al., 2019). The T-DNA cassette can also be transferred into protoplasts by PEG-mediated transfection or electroporation. Alternatively, magnetofection and electroporation transformation methods transferring the T-DNA cassette to microspore and pollen have been used for cotton and wheat genome editing (Figure 3A) although the transformation efficiency is extremely low (Zhao et al., 2017; Bhowmik et al., 2018; Vejlupkova et al., 2020). Recently, simple and high-throughput T-DNA binary vector cloning systems, which can express CRISPR-Cas reagents, including SpCas9 and a sgRNA or multiplexed gRNAs under the control of *Cauliflower Mosaic Virus* (*CaMV*) *35S* and *U6*/*U3* promoters, have been developed for plant genome editing (Kim et al., 2016; Oh et al., 2020), and transformation efficiencies have been increased using nanoparticles to deliver DNA to plant cells (Jat et al., 2020). However, regeneration of transformants remains a limiting factor.

Plant virus-mediated genome editing is highly efficient and avoids the tissue regeneration steps since viruses replicate and systemically move around in plants (Figure 3B). However, because the CRISPR-Cas9 system is too large to be integrated into the genome of viruses commonly used for plant gene function studies and/or to be delivered into plant cells by infection of viruses, virus-mediated genome editing in plants should be conducted in *Cas9*-overexpressing plants. Single-stranded RNA viruses, such as tobacco rattle virus (Ali et al., 2015, 2018), tobacco mosaic virus (TMV; Cody et al., 2017), pea early-browning virus (PEBV; Ali et al., 2018), barley stripe mosaic virus (BSMV; Hu et al., 2019), foxtail mosaic virus (FoMV; Mei et al., 2019), beet necrotic yellow vein virus (BNYVV; Jiang et al., 2019), and the single-stranded DNA (ssDNA) cabbage leaf curl virus (CLCV; Yin et al., 2015), were used for delivering sgRNAs and caused intended sequence modification with high efficiency (up to 80%). Alternatively, virus-mediated genome editing has also been conducted in tobacco (*Nicotiana benthamiana*) plants using two plant negative-stranded RNA (NSR) viruses, barely yellow striate mosaic virus (BYSMV) and sonchus yellow net rhabdovirus (SYNV) that carry Cas9 and sgRNA cassettes simultaneously, and genome-edited lines were generated successfully (Gao et al., 2019; Ma et al., 2020).

Pathways for genome-editing avoiding plant regeneration include transformation of pollen with CRISPR-Cas9 constructs, which has been successfully tested in cotton (Lei et al., 2021), or *de novo* induction of meristems. De novo induction of meristems has been achieved by using morphogenic regulators (MRs) in plants, and it possesses the potential to produce transgenic plants without the need for a tissue culture procedure (Figure 3C). In general, since plant cells are totipotent and can be trans-differentiated into other cell types, ectopic expression of MRs in somatic cells has the potential to induce meristems. Recently, *Agrobacterium* harboring sgRNA and *MR* genes, such as *WUSCHEL2* (*WUS2*), *ISOPENTENYL TRANSFERASE* (*IPT*), and *SHOOTMERISTEMLESS* (*STM*), was inoculated on the *Cas9*-overexpressing tobacco (*N. benthamiana*) plants through pruned sites of plant, and *de novo* induced meristems from the pruned sites eventually became gene-edited plants (Maher et al., 2020). This delivery system can be applied to various plant species requiring a long tissue culture period in generating transgenic plants.

For the CRISPR-Cas9 transfection to plant cells, two systems – transient or stable expression of CRISPR-Cas9 machinery – can be taken into account based on the time frame and goal of the experiment. In RNP and viral delivery systems, components of the CRISPR-Cas9 system do not get incorporated into the plant genome and the target DNA cleavage engaged by the CRISPR-Cas9 takes place for limited period of time. One of the advantages of transient expression systems is reducing the incidence of off-target events due to the limited editing time. Stable expression of CRISPR-Cas9 agents can be also achieved by using a viral vector, in which Cas9 and/or sgRNA are packaged, resulting in permanent insertion into the cellular genome, and this often involves co-transfecting a selectable antibiotic resistant marker gene for selecting plants with the successful genomic integration. Stable transfections are usually applied for long-term expression of CRISPR-Cas9 agents.

## Application of Genome Editing to Crop Breeding

Genome editing, in particular using CRISPR-Cas9 systems, has been extensively applied to various crops. In most cases, it has been used for complete knockout by specific indel mutations to identify the function of the target genes or produce crops with desired traits (Erpen-Dalla Corte et al., 2019). For example, many genes mainly affecting rice quality, yield, and disease resistance have been modified by CRISPR-Cas9 tools (Li et al., 2016b; Lu and Zhu, 2017; Tang et al., 2017; Fiaz et al., 2019). The *Waxy* gene (*Wx*) encoding a granule-bound starch synthase (GBSS) in rice was modified with the CRISPR-Cas9 system to reduce the amylose content to improve grain quality (Zhang et al., 2018b). Xu et al. (2016) also simultaneously mutated *GW2*, *GW5*, and *TGW6* that negatively regulate rice grain weight by using the CRISPR-Cas9-mediated multiplex genome-editing system (Xu et al., 2016). The critical residues, NL within the SVLFPNLAGKS sequence of eukaryotic translation initiation factor 4 gamma responsible for the resistance to rice tungro spherical virus, were identified by CRISPR-Cas9-mediated targeted mutagenesis (Macovei et al., 2018). In addition, intron-targeted site-specific gene replacement using CRISPR-Cas9 tools has also been reported in the rice *endogenous 5-enolpyruvylshikimate-3-phosphate synthase* (*OsEPSPS*); the entire exon 2 of the gene was replaced with a mutated version of the exon containing T102I + P106S amino acid substitutions (Li et al., 2016a). The *OsEPSPS* mutation caused by the amino acid substitutions conferred resistance to glyphosate.

Maize is one of the most important crops in the world. It is used as a model plant for fundamental genetic research due to its tremendous phenotypic and genotypic diversity (Liu et al., 2020). Feng et al. (2015) introduced a sgRNA-Cas9 construct targeting the *Zmzb7* that encodes the IspH protein essential for the methyl-D-erythritol-4-phosphate pathway into maize protoplasts (Feng et al., 2015). Indel mutations were detected in regenerated seedlings, and one seedling showed an expected albino phenotype through screening 120 seedlings generated from 10 callus events. Furthermore, CRISPR-Cas9 has also been utilized to create waxy corn hybrids by editing the *Wx* gene in elite inbred maize lines more quickly compared to the conventional trait introgression process. Four CRISPR-*wx* hybrids displayed a significant increase in grain yield relative to their counterparts (Gao et al., 2020).

In addition, the results of genome editing using the CRISPR-Cas system in tetraploid potato have been released; increased amylopectin/amylose ratio (starch quality) was observed in the line containing all four mutated alleles of the *GBSS* gene with the knockout of GBSS enzyme activity (Andersson et al., 2017). Nakayasu et al. (2018) targeted the *St16DOX*, encoding a steroid 16α-hydroxylase in steroidal glycoalkaloids (SGAs) biosynthesis through CRISPR-Cas9 in order to reduce the level of potato SGAs conferring a bitter taste on human and toxicity against various organisms (Nakayasu et al., 2018). The production of SGA-free hairy roots of tetraploid potato was achieved by using a vector expressing multiplex gRNAs based on the pre-tRNA processing system.

Recently, highly efficient targeted mutagenesis was successfully accomplished by SpRY, an engineered SpCas9 at relaxed PAM sites in a coniferous tree, the Dahurian larch (protoplasts), and in rice transgenic lines (Ren et al., 2021). The case studies clearly demonstrated that the engineered Cas9 was able to break PAM restriction barriers, allowing researchers to edit anywhere in the genomic sites.

In cases where large deletions yield the desired phenotype, CRISPR-Cas9 mutagenesis targeting multiple sites nearby can be a useful strategy. Four sgRNAs targeting the tomato *PMR4* gene, a callose synthase implicated in papillae formation in response to powdery mildew infection over a sequence interval of about 3,700 bp resulted in deletions of up to 900 bp (Santillán Martínez et al., 2020).

Coding sequences were not always targeted by CRISPR-Cas9 mutagenesis. Targeted mutagenesis of promotor sequences was successfully used to alter the expression of target genes (Li et al., 2020a). Manipulating gene expression through promotor mutagenesis is particularly useful for genes, where expression changes yield the desired phenotypes and, also for the case that knockouts cause a lethal phenotype, as they have an essential function.

## New Breeding Strategies for Vegetables with Improved Traits: Case Studies

Vegetables are recognized as essential crops for the human diet due to their rich nutrient content and phytochemicals that contribute to disease prevention and maintenance of health. Recent research recommended consumption of over 400 grams of vegetables and fruits per day to reduce the risk of being affected by cardiovascular disease or cancer and maintain good health (Aune et al., 2017). Vegetable crops are prone to damage by numerous pests and diseases caused by viruses, bacteria, and fungi, and they are also susceptible to abiotic stresses, such as drought, salinity, flooding, and nutrient deficiency (Hodges and Toivonen, 2008; Boscaiu and Fita, 2020). Therefore, the development of varieties that are resistant to biotic stresses and tolerate abiotic stresses and at the same time have good yields and high nutrient content is the goal of most breeding programs. CRISPR-Cas9 is a candidate technology that can help to reach this goal (WHO, 2003; Karkute et al., 2017; Khatodia et al., 2017).

However, there are many challenges on the way to apply CRISPR-Cas systems in vegetable breeding. First, the genomes of most vegetable species, with the notable exception of tomato, are much less researched than the genomes of staple crops, making the selection of mutagenesis targets more difficult (Li et al., 2019). Accordingly, whole-genome sequence information, well-annotated genes, and functional genomic information are essential to successfully identify the candidate genes for mutagenesis. Second, many traits aimed at by breeders are multigenic, while CRISPR-Cas9 is mostly suitable to target one or a few genetic loci, limiting its application. However, the experiments suggest that site-directed large chromosome rearrangements and translocations are possible (Beying et al., 2020), and these modifications concern many genes, making these approaches more useful to manipulate many linked genes at once. And third, generation of mutants with loss-of-function is much easier than those with gain-of-function by CRISPR-Cas9 mutagenesis, representing a further limitation of the technology. Nevertheless, targeting genes with a major function in the trait of interest may result in an improved phenotype.

Based on previously published research articles, information related to the genome editing by CRISPR-Cas systems in vegetables has been collected (Table 1).

**Table 1:** Application of CRISPR-Cas9-based editing of genes in vegetables.

Vegetable		Target gene	Modification/mutant trait	Delivery method	Reference
Fruit vegetables	Tomato	*SIAGO7*	KO/leaflets lacking petioles and later-formed leaves lacking laminae	*Agrobacterium*-mediated transformation	Brooks et al., 2014
		*RIN*	KO/incomplete-ripening fruits	*Agrobacterium*-mediated transformation	Ito et al., 2015
		*DELLA*, *ETR1*	Substitutions/marker gene-free plants harboring stable DNA substitutions	*Agrobacterium*-mediated transformation	Shimatani et al., 2017
		*SLMAPK3*	KO/reduced drought tolerance	*Agrobacterium*-mediated transformation	Wang et al., 2017
		*PROCERA*	KO/derepressed growth	*Agrobacterium*-mediated transformation	Tomlinson et al., 2019
		*SlCLV3*	Mutation in *SlCLV3* promoter/phenotypic changes in fruit size, flower morphology, and locule number	*Agrobacterium*-mediated transformation	Rodríguez-Leal et al., 2017
		*SP*, *SP5G*, *SlCLV3*, *SlWUS*	Gene editing of coding sequences, *cis*-regulatory regions, or upstream open reading frames (ORF)/*de novo*-domesticated tomato	*Agrobacterium*-mediated transformation	Li et al., 2018b
		*SP*, *O*, *FW2.2*, *CycB*, *FAS*, *MULT*	Simultaneous CRISPR–Cas9 editing of six genes/modification of fruit number, size, shape, nutrient content, and plant architecture	*Agrobacterium*-mediated transformation	Zsögön et al., 2018
	Cucumber	*eIF4E*	KO/resistance to ipomovirus, potyviruses zucchini yellow mosaic virus and papaya ring spot mosaic virus-W	*Agrobacterium*-mediated transformation	Chandrasekaran et al., 2016
		*CsWIP1*	KO/gynoecious phenotype	Enhanced *Agrobacterium*-mediated transformation using vacuum infiltration	Hu et al., 2017
	Watermelon	*ClPDS*	KO/albino phenotype	*Agrobacterium*-mediated transformation /PEG-mediated protoplast transfection	Tian et al., 2017
		*ClALS*	Point mutation/herbicide resistance	*Agrobacterium*-mediated transformation	Tian et al., 2018
		*ClPSK1*	KO/enhanced resistance to *Fusarium oxysporum* f. sp. *niveum*	*Agrobacterium*-mediated transformation	Zhang et al., 2020b
		*ClWIP1*	KO/gynoecious watermelon	*Agrobacterium*-mediated transformation	Zhang et al., 2020a
	Eggplant	*SmelPPO*	KO/lowered enzymatic browning in eggplant berries	*Agrobacterium*-mediated transformation	Maioli et al., 2020
Leafy vegetables	Lettuce	*LsBIN2*	KO/targeted gene disruption in whole plants regenerated from protoplasts	PEG-mediated protoplast transfection	Woo et al., 2015
		*LsNCED4*	KO/loss of thermoinhibition	*Agrobacterium*-mediated callus or somatic explants transformation	Bertier et al., 2018
		*LsGGP2*	Deleted uORFs of *LsGGP2* for increasing the translation of mRNAs/increased oxidation stress tolerance and ascorbate content	*Agrobacterium*-mediated callus or somatic explants transformation	Zhang et al., 2018a
	Chicory	*CiPDS*	KO/albino phenotype	*Agrobacterium*-mediated transformation/PEG-mediated protoplast transfection	Bernard et al., 2019
	Chinese kale	*BaPDS1*, *BaPDS2*	KO or KD/albino phenotype	*Agrobacterium*-mediated transformation	Sun et al., 2018
		*BoaCRTISO*	KD/yellow color of Chinese kale with improved market prospects	*Agrobacterium*-mediated transformation	Sun et al., 2020
	Cabbage	*BoPDS*	KO/albino phenotype	*Agrobacterium*-mediated transformation	Ma et al., 2019a
				Electro-transfection in RNP delivery to protoplast	Lee et al., 2020
		*BoPDS1*, *BoSRK3*, *BoMS1*	Multisite and multiple gene KO using an array of sgRNA-tRNA/male-sterile line	*Agrobacterium*-mediated hypocotyls transformation	Ma et al., 2019b
	Chinese cabbage	*BraFLCs*	KO/early-flowering phenotype that did not depend on vernalization	*Agrobacterium*-mediated transformation	Jeong et al., 2019

### Fruit Vegetables

#### Tomato

Tomato (*Solanum lycopersicum* L.), owing to the readily available genomic resources and its global economic importance, is the representative vegetable crop where CRISPR-Cas9 approaches have been tested for crop improvement. In this species, the first report on CRISPR-Cas9-mediated genome editing was in 2014, targeting the *SIAGAMOUS-LIKE 6* (*SIAGL6*) gene responsible for leaf development (Brooks et al., 2014). The CRISPR-Cas9 system was also applied to induce targeted mutations in an exon and an untranslated region (UTR) of *RIPENING INHIBITOR* (*RIN*) gene, which encodes a MADS-domain transcription factor regulating tomato fruit ripening (Ito et al., 2015). The mutant showed an incomplete ripening phenotype, indicating the importance of functional roles and potential applications of *RIN* in tomato fruit. Shimatani et al. (2017) utilized a target activation-induced cytidine deaminase (Target-AID; CRISPR-AID) composed of a nCas9 (D10A) and *Petromyzon marinus* cytidine deaminase 1 (PmCDA1) together with sgRNAs to produce marker-free plants with homozygous heritable DNA substitutions in two endogenous tomato genes, *DELLA* (Solyc11g011260) or *Ethylene Resistance 1* (*ETR*1; Solyc12g011330) that regulate plant hormone signaling (Arazoe et al., 2017; Shimatani et al., 2017). Moreover, tomato plants with CRISPR-Cas9-mediated *slmapk3* (*Solanum lycopersicum mitogen-activated protein kinase 3*) mutation exhibited enhanced tolerance to heat stress: They showed less severe wilting, milder membrane damage with lower reactive oxygen species (ROS) contents, and presented higher levels of both activity and transcript abundance of antioxidant enzymes under the heat stress condition (Yu et al., 2019). In 2019, Tomlinson et al. reported the first use of CRISPR technology to create a dominant dwarf mutation, *PRO^D^* by modifying the tomato *PROCERA* gene that encodes a DELLA protein, a key negative regulator of gibberellin signaling (Yoshida et al., 2014). They analyzed phenotypes of heterozygous and homozygous mutant plants for *PRO^D^*: *PRO^D^/PRO^D^* and *PRO^D^/PRO*. At the seedling stage, *PRO^D^/PRO* plants exhibited an intermediate phenotype in plant height between WT (*PRO/PRO*) and homozygous (*PRO^D^/PRO^D^*) plants indicating that the mutation is semidominant at that stage. Later in development, however, both heterozygous and homozygous plants for *PRO^D^* were equally dwarfed compared to WT (Tomlinson et al., 2019). Compared to mutations in the coding region to create null alleles, the advent of CRISPR-Cas9-mediated engineering in cis-regulatory elements (CREs) required for the expressional regulation of a coding sequence provides a more refined method for creating phenotypic diversity for enhancing crop breeding (Swinnen et al., 2016; Wolter and Puchta, 2018). Indeed, multiplexed CRISPR-Cas9 mutagenesis in the *SlCLV3* promoter region created a series of cis-regulatory alleles, resulting in a range of changes from the transcriptional level of *SlCLV3* to phenotypes in fruit size, flower morphology, and locule number. Hence, this approach offers the possibility of more efficient breeding by fine-tuning the expression of genes associated with improved yield and crop quality (Rodríguez-Leal et al., 2017).

The capacity to simultaneously target multiple genes for editing facilitated an experiment to perform some major steps in “redomestication” of tomato. Through CRISPR-Cas9-mediated disruption of six independent genes in wild tomato (*Solanum pimpinellifolium*), fruit size and fruit number in mutated wild tomato were increased three- and ten-fold, respectively (Zsögön et al., 2018). Of note, the “redomesticated” tomato with improved fruit and agronomic traits retained the increased tolerance to salt stress and disease (Li et al., 2018b). Besides these approaches aiming at phenotypic changes, CRISPR-Cas9 became a routine technology for functional genomics, including validation of candidate genes identified in genome-wide association studies (Alonge et al., 2020).

#### Cucumber

Cucumber (*Cucumis sativus* L.), belonging to the *Cucurbitaceae* family, is also an economically important vegetable (Huang et al., 2009) that is cultivated in nearly all countries within both temperate and tropical zones (Tatlioglu, 1993). The first application of CRISPR-Cas9 in cucumber was for conferring broad viral resistance through knockout of *eukaryotic translation initiation factor 4E* (*eIF4E*) gene (Chandrasekaran et al., 2016). Gynoecious inbred lines in cucumber have great importance due to their higher production yield and the lower labor cost required for crossing (Robinson, 2000). Hu et al. (2017) generated *Cswip1* mutants by CRISPR-Cas9 tools, targeting the *WPP trp/pro/pro domain Interacting Protein1* (*CsWIP1*) gene, which encodes a zinc-finger transcription factor. *Cswip1* T0 mutants exhibited gynoecious phenotype bearing only female flowers, implying that the gene is involved in inhibition of cucumber carpel development (Hu et al., 2017).

#### Watermelon

Watermelon (*Citrullus lanatus*), which belongs to the *Cucurbitaceae* family, is a rich source of citrulline, vitamins, and lycopene (Collins et al., 2007; Maoto et al., 2019). CRISPR-Cas9-mediated mutations in the *phytoene desaturase* (*ClPDS*) gene, encoding a key enzyme of carotenoid synthesis, caused the expected albino phenotype in watermelon plants (Tian et al., 2017). The CRISPR-Cas9-mediated base editing system was also utilized to achieve single-nucleotide conversion at the *acetolactate synthase* (*ClALS*) gene in watermelon (Tian et al., 2018). Watermelon plants possessing C to T mutations in the codon of Pro 190 (CCG) at the *ClALS* gene have become resistant to all sulfonylurea herbicides without compromising fruit and seed size, and seed yield (Yu and Powles, 2014). In addition, the CRISPR-Cas9 system was used to generate the knockout mutation of the *phytosulfokine1* (*ClPSK1*) gene responsible for the infection by *Fusarium oxysporum* f. sp. *niveum* (*FON*). The loss-of-function mutation of *ClPSK1* rendered watermelon seedlings more resistant to infection by *FON* (Zhang et al., 2020b). Recently, it was reported that the *ClWIP1*, a homologue of *CsWIP1* and *CmWIP1* in cucumber and melon, respectively, acts as the gynoecious (*gy*) gene in watermelon (Martin et al., 2009). The *ClWIP1* is specifically expressed in carpel primordia in male floral buds and also linked to the abortion of carpel primordia in early floral development. Artificial gynoecious watermelon lines have been generated using the CRISPR-Cas9 system targeting the *ClWIP1* (Zhang et al., 2020a).

#### Eggplant

Eggplant (*Solanum melongena* L.) ranks fifth among vegetables in terms of total global production, with 52.3 million tons produced in 2017 (Alam and Salimullah, 2021). Three *polyphenol oxidase* genes (*PPOs*; *SmelPPO4*, *SmelPPO5*, and *SmelPP*O6) showing the highest transcript levels in the fruit after cutting were regarded to be associated with enzymatic browning of eggplants, and CRISPR-Cas9-based mutagenesis has been applied to knockout three target *PPO* genes simultaneously aiming to reduce fruit flesh browning (Maioli et al., 2020).

### Leafy Vegetables

#### Lettuce

Leafy vegetables have also been edited for trait improvement through CRISPR-Cas applications. In particular, a DNA-free genome-editing approach has been applied in lettuce and cabbage via Cas9- and Cpf1-RNPs delivery into protoplasts. Woo et al. (2015) succeeded in delivering CRISPR-Cas9 RNPs into lettuce protoplasts by PEG-mediated transfection and, subsequently, regenerated plants with intended mutations from the protoplasts (Woo et al., 2015). Recently, it has been suggested that electroporation is more efficient in RNP delivery to protoplasts than PEG-mediated transfection in cabbage based on the results of *phytoene desaturase1* (*PDS1*) sgRNA delivery, which may be due to lower chemical toxicity (Lee et al., 2020). It has also been found that genome editing of upstream open reading frame (uORF) enabled the modulation of translation of mRNA. Editing the uORF of *LsGGP1* and *LsGGP2*, which encodes a key enzyme in vitamin C biosynthesis, increased mRNA translation, thereby elevating ascorbate content and oxidation stress tolerance.

#### Other Leafy Vegetables

Like other vegetables, the CRISPR-Cas9-based gene knockout or knockdown was applied to *PDS* genes in chicory (*Cichorium intybus* L.; Bernard et al., 2019), Chinese kale (*Brassica oleracea* var. *alboglabra*), and cabbage (*Brassica oleracea* var. *capitata*; Ma et al., 2019a,b; Lee et al., 2020), and *PDS* mutants were detected by their albino phenotype. CRISPR-Cas9 was also used to induce mutations in the *carotenoid isomerase* gene (*BoaCRTISO*) of Chinese kale (Sun et al., 2020). The color of biallelic and homozygous *CRTISO* mutants was changed from green to yellow in the leaves and bolting stems. Also, Jeong et al. (2019) succeeded in generating the early flowering Chinese cabbage (*Brassica rapa* spp. *pekinensis*) by the CRISPR-Cas9-mediated knockout in *FLOWERING LOCUS C* (*BraFLC*) genes homologous to *Arabidopsis FLC*, which encodes a MADS domain protein that plays a central role in repressing flowering (Deng et al., 2011; Jeong et al., 2019).

## Perspective

The CRISPR-Cas systems confer significant opportunities for improvement in crop production by mitigating biotic and abiotic stresses as well as increasing yield (Doudna and Charpentier, 2014; Langner et al., 2018). Many consumers believe that the production of genetically modified organisms (GMOs) is risky for the environment and health and that it may be dangerous to consume GMOs mostly due to lack of consumer knowledge about GMOs (Wunderlich and Gatto, 2015). However, gene editing using CRISPR-Cas9 systems differs noticeably from the conventional genetic modification in the 1990s. While conventional genetic modification introduces foreign DNA into a crop, CRISPR, like mutation breeding, only induces small changes in the native DNA, and in contrast to mutation breeding, these changes are not random but generally well defined (Shew et al., 2018). But in any case, plants released into the environment must be transgene free as CRISPR gene-editing constructs are potentially hazardous to the environment (Huang et al., 2016). Furthermore, traits in commercial crops must be stable, and a functioning CRISPR-Cas9 system in plants may cause phenotypic variation through off-target mutations (He and Zhao, 2020). In sexually propagated plants, the CRISPR-Cas9 cassette could be eliminated by crosses and marker-assisted selection of segregants carrying the mutation but not the CRISPR-Cas9 cassette, or transgene-free cells can be selected expressing suicide genes in a temporary-controlled manner.

Off-target mutagenesis through CRISPR-Cas9 is considered a risk for medical approaches in humans and animals, but much less so in plants, where suitable phenotypes can be selected from a large number of mutant genotypes. Moreover, recent results clearly indicate that off-target effects through genome editing occur by orders of magnitude less frequently, compared to chemical or irradiation methods to induce mutations in plants (Modrzejewski et al., 2020).

Nevertheless, analyses methods for nontarget mutations are becoming more efficient (Liu et al., 2021a), and tools available for animal research could also be adapted for plants to assess the unexpected effects of genome editing (Liu et al., 2021b). Generally, one of the two global regulatory policies for genome-editing system has been used in different countries (i.e., product based and process based; Globus and Qimron, 2018). The United States (Kleter et al., 2019), Argentina (Gao et al., 2018), and many other countries have adopted a product-based approach, which evaluates the safety of genome-modified end-products, while the European Union (Jouanin et al., 2018) and New Zealand (Fritsche et al., 2018) have chosen to regulate genome-edited products based on the process used. And if recombinant DNA technology is used to produce the mutated plant, according to a process-based regulation, it is considered to be a genetically modified organism. According to these regulatory policies, genome-edited plants through CRISPR-Cas9 systems have been differently classified as GMOs or non-GMOs in different countries. CRISPR-Cas9 regulation is not finalized in most countries; some countries classified edited plants without a transgene [the so-called site directed nuclease technology-1 (SDN-1) events] as non-GMO, while other countries consider edited plants as GMO (Zhang et al., 2020c). SDN-2 events, like SDN1 events, are produced with a DSB, but for repair of the break, a small nucleotide template complementary to the area of the break is provided to cause the insertion of a short sequence into the gap. SDN-2 events, in the strict sense, do not contain foreign DNA (except a few additional bases) and therefore are not considered as GMO in most countries with a product-based gene-editing regulation. SDN-3 events are analogously produced, like SDN-2 events, but the template provided to guide the repair contains a gene or other sequence. SDN-3 plants therefore are generally considered as GMO.

There is currently ongoing controversy about the regulation of new gene-editing techniques because the strict regulatory policies, especially the regulatory framework in the EU, will restrict the application of genome-editing technology which has tremendous potential for improving crops although a very recent report from the European Commission presented a positive viewpoint on innovation in gene editing with maintaining a cautionary tone (Friedrich, 2020).^1^ More efficient and robust gene-/genome-editing systems, including the components of their delivery systems with fewer off-target effects for plant species, will be updated continuously. Particularly, multiple omics approaches, such as whole genome sequencing, RNA/small RNA-seq, and proteome analyses, will be of great help in finding more target genes for gene-/genome-editing-based vegetable improvement because selecting/finding appropriate genes for a desired trait is the key for the ultimate generation of improved vegetables through precise manipulation of genetic elements required for phenotypic alterations, particularly for the traits with a complicated genetic background, such as yield and quality. Thus, continuous efforts to identify genetic players that directly or indirectly control desirable traits and serve as targets for genome editing in vegetables are also required.

## Author Contributions

All authors listed have made a substantial and intellectual contribution to the work and approved the final version of the manuscript for publication.

### Conflict of Interest

The authors declare that the research was conducted in the absence of any commercial or financial relationships that could be construed as a potential conflict of interest.

## Footnotes

^1^https://ec.europa.eu/food/sites/food/files/plant/docs/gmo_mod-bio_ngt_eu-study.pdf
